# Egbert’s Hygiene and Sanitation

**Published:** 1899-04

**Authors:** 


### Books and Magazines.

EGBERT'S HYGIENE AND SANITATION.—A Manual of Hygiene and Sanitation. By Seneca Egbert, A. M., M. D., Professor of Hygiene in the Medico-Chirurgical College of Philadelphia. In one handsome 12mo. volume of 360 pages with 63 engravings. Cloth, $2.25 net. Lea Brothers & Co., Publishers, Philadelphia and New York.

A concise, complete and thorough manual, upon a subject upon which I suspect we Texas doctors need some enlightenment occasionally. If Hygiea should make a sudden demand for attentive worship at her altars I fear some of us would prove to be but indifferent priests, notwithstanding our loud professions of devotion. Seriously I am convinced that, with the exception of those who have had the advantages of a university education in recent years, physicians have but a diffuse and hazy knowledge of even the general principles of hygiene and sanitation. If called upon to give an extemporaneous opinion upon a subject involving the ordinary hygiene of daily life, we usually contradict each other to such an extent that our opinions are either not sought or disregarded, and it would be hard to convince the public that they are of sufficient value as to merit a fee. This, I am convinced, has arisen, not so much from a lack of diligence in our studies, but from the fact that the literature that we have studied is either so general and diffuse in its statements as not to convey practical information, or as is mostly the case, it is of experimental character, and is usually contradicted by the next author we read.

This leads to the statement that I desire to make about the work under consideration. After a careful consideration I fail to find a statement in it that is now *sub judice*. It is a concise, exact manual of sanitary science, so far as it is understood, and contains just the information for which we frequently search the pages and indices of more pretentious works in vain.

The titles discussed are: Bacteriology, The Atmosphere—Air, Ventilating and Heating, Water, Food, Stimulants and Beverages Personal Hygiene, School Hygiene, Disinfection and Quarantine, The Removal and Disposal of Sewage, Vital Statistics, The Examination of Air, Water and Food. The work is strongly recommended. I. J. J.

